# Page for the General Public

**DOI:** 10.1055/s-0038-1654698

**Published:** 2018-07-27

**Authors:** Anneke Damberg

**Affiliations:** 1 AORTA Journal Editorial Office, New Haven, Connecticut

The following pages summarize and review this issue's articles for an audience without a background in medicine or research.

1. Yvan Devaux et al.: “Epigenetics in Ascending Thoracic Aneurysm and Dissection”
  Aortic aneurysm and aortic dissection are potentially life-threatening diseases of the aorta, the body's main artery. Both diseases share a genetic predisposition which causes a weakness of the vessel wall. The authors of the article discuss new findings regarding the “epigenetics” of these diseases. Epigenetics refers to how cells read the genetic codes, for example, if a genetic code is read at all and how much of the molecule it stands for is produced. The authors suggest that these mechanisms play an important role in the development of aortic diseases, but they are not yet very well understood. Epigenetic mechanisms could be a target for future diagnostic and therapeutic approaches.
2. Adam Brownstein et al.: “Genes Associated with Thoracic Aortic Aneurysms and Dissection: A 2018 Update and Clinical Implications”
  Thoracic aortic aneurysm and dissection are diseases of the vessel wall that can cause rupture of the body's main artery, the aorta. Over the past decade, over 30 genes have been found that are associated with these diseases. In about every third patient, one of these genes can be found. The authors give an update of the most recent findings, including the current understanding as to when surgical treatment should be considered in patients with the respective genes. With increasing knowledge of the genetics of aortic diseases, a more personalized treatment might be possible.
3. Claudio Bianchini Massoni et al.: “Ruptured Abdominal Aneurysm in a Patient with Congenital Fused Pelvic Kidney: A Case Report of Emergency Endovascular Treatment”
  In some patients, the kidneys are abnormally located or connected to each other. These patients often have more or abnormally located vessels leading to the kidneys which need to be taken into account when planning procedures on the abdominal aorta, the body's main artery in the abdomen. The authors describe a case of a patient and discuss treatment options and important aspects in procedural planning in patients with abdominal aortic disease and kidney anomalies.
4. Mariano Camporrotondo et al.: “Surgical Treatment of Dysphagia Lusoria Caused by Right-Aortic Arch with Kommerell Diverticulum. Left Heart Bypass without Subclavian Revascularization.”
  Camporrotondo and colleagues describe a case of a young patient with an anomaly of the aortic arch, a part of the body's main artery in the chest. This anomaly caused a vessel to compress the esophagus, which led to discomfort during swallowing in the patient. The authors describe their experience and discuss different treatment options.
5. Spyridon N. Vasdekis et al.: “An Unusual Case of Acute Thrombosis of Abdominal Aortic Aneurysm without Acute Limb Ischemia”
  In patients with abdominal aortic aneurysm, an abnormal dilatation of the body's main artery in the abdomen, blood clots can form in the vessel and cause obstruction. Often, this obstruction becomes apparent by acute blockage of the blood flow to the legs. The authors describe a case of a patient with obstruction of the abdominal aorta due to a blood clot who had no symptoms. The authors discuss risk factors for abdominal aneurysm thrombosis and treatment options.
6. Fabio Ramponi et al.: “Successful Repair of Concomitant Acute Type A Dissection and Saddle Pulmonary Embolism”
  Acute aortic dissection and pulmonary embolism are both acute life-threatening emergencies. In acute aortic dissection, a tear develops in the aorta, the body's main artery. In pulmonary embolism, a blood clot blocks the lung vessels. The authors describe a case of a patient who presented with acute chest pain and turned out to have both directions. The authors describe how they performed surgery to repair both diseases and discuss important aspects in diagnosing and differentiating aortic dissection and pulmonary embolism.
7. Maude Cameron-Gangné et al.: “Buttocks Hard as Rocks: Not Wanted after Aortic Dissection Repair”
  The authors describe a case of a patient with acute aortic dissection, a tear in the body's main artery. He underwent emergency surgery. After the procedure, he developed severe pain in his buttocks due to “compartment syndrome.” In compartment syndrome, the pressure in a muscle compartment increases to dangerous levels, because blood flow to the muscles is impaired. The muscle compartment had to be urgently surgically opened to release the pressure. The patient recovered well.
8. Murat Ugurlucan et al.: “Anatomic Revascularization of the Celiac Trunk and the Superior Mesenteric Artery”
  When the vessels providing blood flow to the bowel are calcified, this can cause severe abdominal pain, weight loss, and even gangrene. To restore blood flow, minimally invasive techniques and open surgical techniques exist. The authors describe their surgical technique in a patient who underwent open surgery in which a vein from the leg was used to bypass the calcified vessels. The patient recovered without any complications.
9. Murat Ugurlucan et al.: “Endovascular Thoracoabdominal Replacement after Total Abdominal Aortic Debranching”
  Marfan's syndrome is an inherited disease of the connective tissue that often affects the aorta, the body's main artery. In many patients, parts of the aorta need to be replaced during their lifetime. Ugurlucan and colleagues describe a case of a patient who refused open surgical replacement of the aorta in his abdomen. Instead, the authors performed a procedure in which the vessels providing blood to the abdominal organs were connected to a vessel in the groin. Then, the diseased part of the abdominal aorta was covered from the inside with a stent graft that was introduced through a vessel in the groin as well. This technique is not the first choice in young patients, but it can be an option if the patient has a high surgical risk or refuses the standard procedure.
10. Umberto Rossi et al.: “Right Iliac Artery—Inferior Vena Cava Arteriovenous Fistula”
  An arteriovenous fistula is a connection between an artery (a vessel leading blood from the heart into the body) and a vein (a vessel leading blood back to the heart). Rossi and colleagues discuss a case of a patient who had a dilatation of an artery in his pelvis that ruptured and led to a connection with the body's largest vein, the vena cava. To avoid open surgery, the patient was treated with a stent graft that was inserted into the ruptured artery via an artery in the groin and covered the ruptured segment.
11. Ioannis Dimarakis et al.: “Hemi-Cabrol Aortic Root Replacement in Complex Aortic Reconstruction”
  The first part of the aorta, the body's main artery, where it arises out of the heart, is called aortic root. The coronary arteries, which provide blood flow to the heart muscle, arise from the aortic root. When the aortic root needs to be replaced because of a disease of the aorta, the coronary vessels need to be reimplanted into the prosthesis graft. This procedure can be challenging. The authors discuss a surgical technique called “Cabrol Aortic Root Replacement” that can be used when it is not possible to directly connect the coronary arteries to the graft. In Cabrol Aortic Root Replacement, a small tubed prosthesis is used to connect the coronary vessels to the aortic root. The authors describe two cases in which they used this technique and discuss their experience.

